# Homogeneous Time-Resolved Fluorescence-Based Assay to Monitor Extracellular Signal-Regulated Kinase Signaling in a High-Throughput Format

**DOI:** 10.3389/fendo.2014.00094

**Published:** 2014-06-23

**Authors:** Mohammed Akli Ayoub, Julien Trebaux, Julie Vallaghe, Fabienne Charrier-Savournin, Khaled Al-Hosaini, Arturo Gonzalez Moya, Jean-Philippe Pin, Kevin D. G. Pfleger, Eric Trinquet

**Affiliations:** ^1^Molecular Endocrinology and Pharmacology, Harry Perkins Institute of Medical Research and Centre for Medical Research, The University of Western Australia, Nedlands, WA, Australia; ^2^Cisbio Bioassays, Codolet, France; ^3^Department of Molecular Pharmacology, CNRS UMR5203, INSERM U661, Institute of Functional Genomics, Universities Montpellier 1 & 2, Montpellier, France

**Keywords:** HTRF^®^, ERK1/2, GPCR, RTK, EGFR

## Abstract

The extracellular signal-regulated kinases (ERKs) are key components of multiple important cell signaling pathways regulating diverse biological responses. This signaling is characterized by phosphorylation cascades leading to ERK1/2 activation and promoted by various cell surface receptors including G protein-coupled receptors (GPCRs) and receptor tyrosine kinases (RTKs). We report the development of a new cell-based Phospho-ERK1/2 assay (designated Phospho-ERK), which is a sandwich proximity-based assay using the homogeneous time-resolved fluorescence technology. We have validated the assay on endogenously expressed ERK1/2 activated by the epidermal growth factor as a prototypical RTK, as well as various GPCRs belonging to different classes and coupling to different heterotrimeric G proteins. The assay was successfully miniaturized in 384-well plates using various cell lines endogenously, transiently, or stably expressing the different receptors. The validation was performed for agonists, antagonists, and inhibitors in dose–response as well as kinetic analysis, and the signaling and pharmacological properties of the different receptors were reproduced. Furthermore, the determination of a *Z*′-factor value of 0.7 indicates the potential of the Phospho-ERK assay for high-throughput screening of compounds that may modulate ERK1/2 signaling. Finally, our study is of great interest in the current context of investigating ERK1/2 signaling with respect to the emerging concepts of biased ligands, G protein-dependent/independent ERK1/2 activation, and functional transactivation between GPCRs and RTKs, illustrating the importance of considering the ERK1/2 pathway in cell signaling

## Introduction

The activation of mitogen-activated protein kinases (MAPKs) constitutes one of the major intracellular signaling pathways that couples signals from cell surface receptors, such as receptor tyrosine kinases (RTKs) and G protein-coupled receptors (GPCRs), to gene expression regulation and other intracellular events (1–6). Indeed, following stimulation, these receptors promote a sequential protein kinase cascade (involving MAP4Ks, MAP3Ks, and MAP2Ks) leading to the activation of ERKs (mostly ERK1/2, also called p44/p42 MAPK) by MAP2K members (also called MEKs) (5, 7, 8). In this cascade, MEK1/2 is phosphorylated and activated by the Ras/Raf pathway and other MAP kinase kinase kinases (MAP3Ks) through serine phosphorylation at the typical Ser-Xaa-Ala-Xaa-Ser/Thr motif in their activation loop (serine 218 and 222 for MEK1) (8, 9). Then, the activation of the ERK1/2 occurs by phosphorylation of both threonine and tyrosine residues in the Thr-Glu-Tyr motif in their activation loop (Threonine 202/Tyrosine 204 for ERK1 and Threonine 185 and Tyrosine 187 for ERK2) (8, 10). The activation of the ERK1/2 pathway leads to the modulation of diverse downstream transcription factors (NF-κB, CREB, AP-1, and c-Myc) controlling the expression of the key genes involved in the control of cell division, differentiation and apoptosis (11).

Therefore, the development of a robust, highly sensitive and specific assay to investigate the activation of ERK1/2 is of great interest for both academic and industry researchers and laboratories. Indeed, for many years the most useful and widely utilized method to investigate the ERK1/2 pathway has been based on SDS-PAGE followed by Western blot using anti-Phospho-ERK1/2 antibodies. Even though successful and reproducible, this method has the considerable limitation of being time-consuming, since protein extraction, electrophoresis, protein transfer, and then overnight antibody incubation are required. In addition, western blot analysis is not always quantitative, impairing applicability to high-throughput screening (HTS), and drug profiling programs. Alternative recent techniques have been proposed and developed (12), such as the enzyme-linked immunosorbent assay (ELISA), the Meso-Scale Assay electrochemiluminescence-based method (13), the LICOR infrared fluorescence-based method (14), and TGR BioSciences’ SureFire^®^ assay that uses the AlphaScreen™ technology (PerkinElmer) (15, 16). These have been reviewed collectively (17). Recently, a BRET-based ERK biosensor has also been developed to temporally and spatially assess ERK activation (18).

Here, we describe a homogeneous time-resolved fluorescence (HTRF^®^) cell-based assay compatible with HTS to investigate the intracellular ERK1/2 signaling pathway. HTRF^®^ combines fluorescence resonance energy transfer (FRET) technology with time-resolved (TR) measurement (19, 20). Since its development, this technology has been applied to many antibody-based assays (19), including second messengers (cAMP and IP-One) (21–24), kinases (25), oligomerization of GPCRs (26, 27) and RTKs (28), G protein activation and β-arrestin recruitment (29), ligand-receptor binding (30, 31), cytokines and biomarkers (32), and protein–DNA/RNA interactions (33), and recently ERK1/2 with the calcium-sensing receptor (22). The Phospho-ERK assay has been validated using the two major cell surface receptor families, RTKs and GPCRs, where both agonist-induced ERK1/2 phosphorylation and inhibition by receptor-selective inhibitors/antagonists as well as the comparison with the classical western blot technique were investigated in cell lines endogenously, transiently, or stably expressing the different receptors.

## Materials and Methods

### Cell lines, plasmids, and reagents

We used the HTRF^®^ Phospho-ERK kit (Catalog number 64ERKPEG) recently launched by Cisbio Bioassays. Chinese hamster ovary (CHO) cells stably expressing the different GPCRs were generated by Euroscreen (Gosselies, Belgium), except the CHO–muscarinic M1 receptor (M1) cell line that was kindly provided by Dr. Denis Servent (CEA–DSV, Saclay, France). NIH-3T3, A431, HEK293, HeLa, and SKOV3 cell lines were from American Type Culture Collection (Manassas, VA, USA). HEK293FT cells were from Life Technologies (Burwood, VIC, Australia). All the plasmids coding for the GPCRs used in Figure 4 were generated and/or purchased by Kevin D. G. Pfleger’s laboratory from Missouri S&T cDNA Resource Center^1^ and AT1R and NMU2R plasmids were gifts from Prof. Walter Thomas (University of Queensland, Australia) and Dr. Gary B. Willars (University of Leicester, UK), respectively. All the agonists (EGF, MCP-1, MIP1β, SDF1α, DAMGO, Endorphin-2, Norepinephrine, Isoproterenol, ACEA, PGE2, ITAC, FTY720, VIP, arginine-vasopressin (AVP), carbachol, Ang II, and human Neuromedin U-25), the antagonist CTOP and Pertussis toxin were from Sigma. The anti-Phospho-ERK1/2 antibody used for western blot was purchased from Cell Signaling Technology.

### Cell culture and transient transfections

Cells were maintained at 37°C, 5% CO_2_ in Complete Media (Dulbecco’s modified Eagle’s medium containing 0.3 mg/ml glutamine, 100 IU/ml penicillin, and 100 mg/ml streptomycin; Gibco) supplemented with 10% fetal calf serum (FCS). Transient transfections were carried out using Lipofectamine 2000 (Invitrogen) or GeneJuice (Novagen) according to manufacturer’s instructions. Cells were harvested with 0.05% Trypsin–EDTA (Gibco).

### One-plate protocol of the Phospho-ERK assay

Cells endogenously, transiently, or stably expressing the different receptors were cultured overnight in a 384-well white plate (5000 cells/well) using 8 μl of medium/well. Then 4 μl/well of the 1× stimulation buffer, containing or not the different agonists at the different concentrations as indicated, were added to the cells. After incubation at room temperature, cells were lysed by adding to the stimulation mix 4 μl/well of the supplemented lysis buffer, followed by incubation for 30–45 min at room temperature with shaking. Then 2 μl of anti-ERK1/2-Europium/Terbium Cryptate and 2 μl of anti-Phospho-ERK1/2-d2 antibody solutions prepared in the detection buffer were added. The plate was then incubated for at least 2 h at room temperature before reading the fluorescence emission at 620 and 665 nm using either a Tecan Infinite 500 (Tecan Group Ltd.) or RUBYstar or PHERAstar FS plate reader (BMG Labtech).

### Two-plate protocol of the Phospho-ERK assay

Cells endogenously, transiently, or stably expressing the different receptors were cultured in a 96-well cell culture plate (5–10 × 10^4^ cells/well, which corresponds roughly to 25 μg of proteins). Twenty-four hours after cell transfection, cells were starved overnight in DMEM-serum free medium and then incubated in a total volume of 50 μl/well of the 1× stimulation buffer, containing or not the different agonists at the different concentrations as indicated. Cells were incubated at room temperature before the stimulation mix was removed and cells were then lysed in 50 μl/well of the supplemented lysis buffer for 30–45 min at room temperature with shaking. Then 16 μl of each lysate was transferred into a 384-well small volume white plate and made up to the final volume of 20 μl/well with 2 μl of anti-ERK1/2-Europium/Terbium Cryptate and 2 μl of anti-Phospho-ERK1/2-d2 antibody solutions prepared in the detection buffer. The plate was then incubated for 2 h at room temperature before reading the fluorescence emission at 620 and 665 nm using a Tecan Infinite 500 (Tecan Group Ltd.), RUBYstar, or PHERAstar FS plate reader (BMG Labtech), or an EnVision 2102 plate reader (PerkinElmer).

### Comparison between HTRF^®^-based Phospho-ERK assay and western blot

A431 cells were seeded in 175 cm^2^ flasks. After 48 h incubation, cells were washed with 10 ml PBS and then stimulated for 5 min with 5 ml of EGF diluted at 100 nM in serum free medium. After stimulation, cells were washed with 10 ml PBS and then lysed with 3 ml of 1× HTRF complete lysis buffer for 30 min at room temperature. Following centrifugation, the supernatant was collected, and serial dilutions of lysates (1:2) were performed in 1× HTRF complete lysis buffer. For the HTRF assay, 16 μl of each diluted sample was dispensed in a white 384-well small volume plate and detection was performed as described in the following section. For western blot analysis, 16 μl of each diluted sample was denatured with Laemmeli buffer and proteins were separated on a polyacrylamide gel (NuPAGE Novex 4–12% Bis–Tris Gel). After electrotransfer, PVDF membranes were blocked in 5% non-fat dry milk in Tris-Buffered Saline – 0.1% Tween-20 (TBS-T). After washing steps, membranes were incubated with anti-Phospho-ERK1/2 or anti-total ERK antibodies, diluted at 1 μg/ml in TBS-T with 5% Bovine Serum Albumin (BSA). After overnight incubation at 4°C under gentle agitation, membranes were washed three times with TBS-T, and incubated with HRP-conjugated secondary antibody. After 1 h incubation at room temperature, membranes were washed four times with TBS-T and incubated with SuperSignal West Femto Chemiluminescent Substrate for 5 min at room temperature. Chemiluminescent signal was acquired on a G:BOX imaging system (Syngene, Cambridge, UK).

### Data presentation and analysis

Data are presented as HTRF ratios, which are calculated as the ratio between the emission at 665 nm and the emission at 620 nm (×10,000). The sigmoidal curves were fitted to the dose–response data using non-linear regression [Log (ligand) versus HTRF ratio] by Prism 5 graphing software (GraphPad, La Jolla, CA, USA).

## Results and Discussion

In this study, we have reported validation of the HTRF^®^-based Phospho-ERK assay to investigate the intracellular ERK1/2 signaling pathway, compatible with HTS in 96-well and 384-well plate formats. Epidermal growth factor receptor (EGFR) as a prototypical RTK and various GPCRs were tested in endogenous, stable, and transient expression models. The assay has been validated by studying multiple aspects including kinetics, dose-dependent effects of agonists and inhibitors, phosphorylated versus total ERK1/2, and the implication of heterotrimeric G proteins.

### Principle of HTRF^®^-based Phospho-ERK1/2 assay (Phospho-ERK assay)

**Figure 1:**
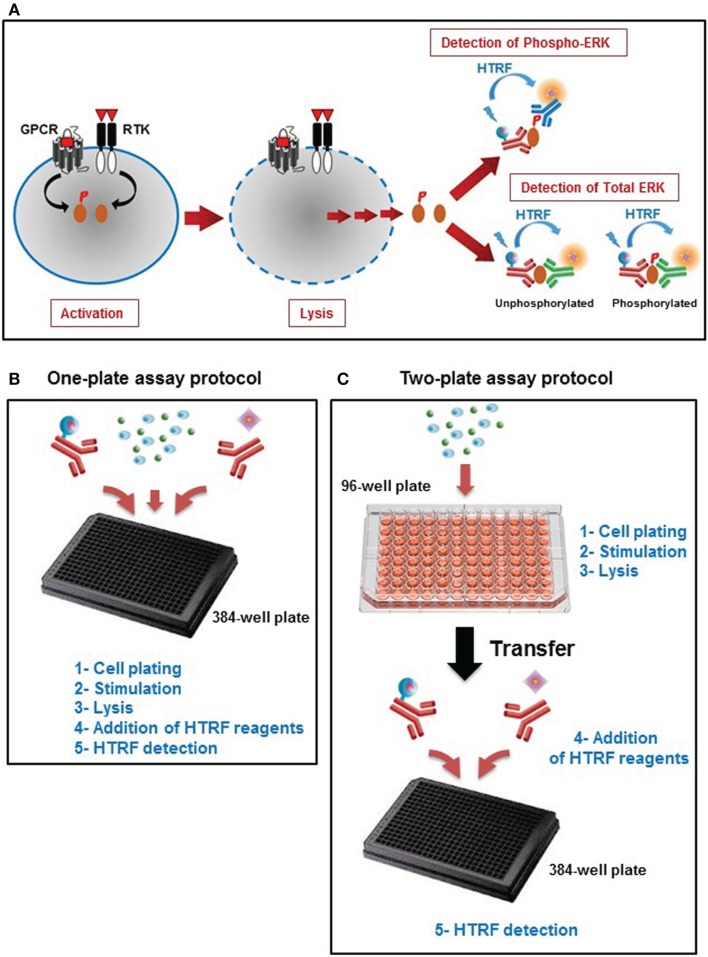
**Principle of the Phospho-ERK assay**. **(A)** Principle of HTRF^®^-based ERK1/2 assay that consists of three experimental steps: activation, cell lysis, and HTRF detection to quantify the total ERK1/2 as well as the phosphorylation of ERK1/2 mediated by the major cell surface receptors. This straightforward assay has been developed with two different protocols: **(B)** the one-plate protocol where all the assay steps are performed in the total volume of 20 μl using one 384-well small volume plate, and **(C)** the two-plate protocol in which the stimulation and lysis steps are performed in the total volume of 50 μl using the initial 96-well plate containing the cells, then the cell lysate is transferred into a 384-well small volume plate for HTRF detection after addition of HTRF conjugated-antibodies as described in Section “Materials and Methods” (Adapted from the CisBio Bioassays website^2^ with permission).

Phospho-ERK is an *in vitro* assay developed to assess the phosphorylation of ERK1/2, a fingerprint of the MAP kinase signaling pathway promoted by a variety of cell surface receptor families such as GPCRs and RTKs. The assay is a sandwich immunoassay comprising three straightforward steps: (i) cell activation, (ii) cell lysis, and (iii) detection of HTRF signals (Figure 1A). The detection of HTRF signals is based on the incubation of the cell lysate with an anti-ERK1/2 antibody labeled with Europium cryptate that recognizes all ERK1/2 proteins, combined with either an anti-ERK1/2 antibody (for the unphosphorylated forms) or anti-Phospho-ERK1/2 antibody (for the phosphorylated forms) labeled with d2 (Figure 1A). The proximity generated by the simultaneous binding of the two antibodies leads to an efficient FRET between the europium cryptate and the sensitized acceptor, d2. The resulting acceptor emission at 665 nm is then used as the assay readout. We have validated the assay using two different protocols, one-plate (Figure 1B), and two-plate (Figure 1C) protocols, as described in Section “Materials and Methods.”

### Use of the Phospho-ERK assay to monitor EGFR-mediated ERK1/2 activation

**Figure 2:**
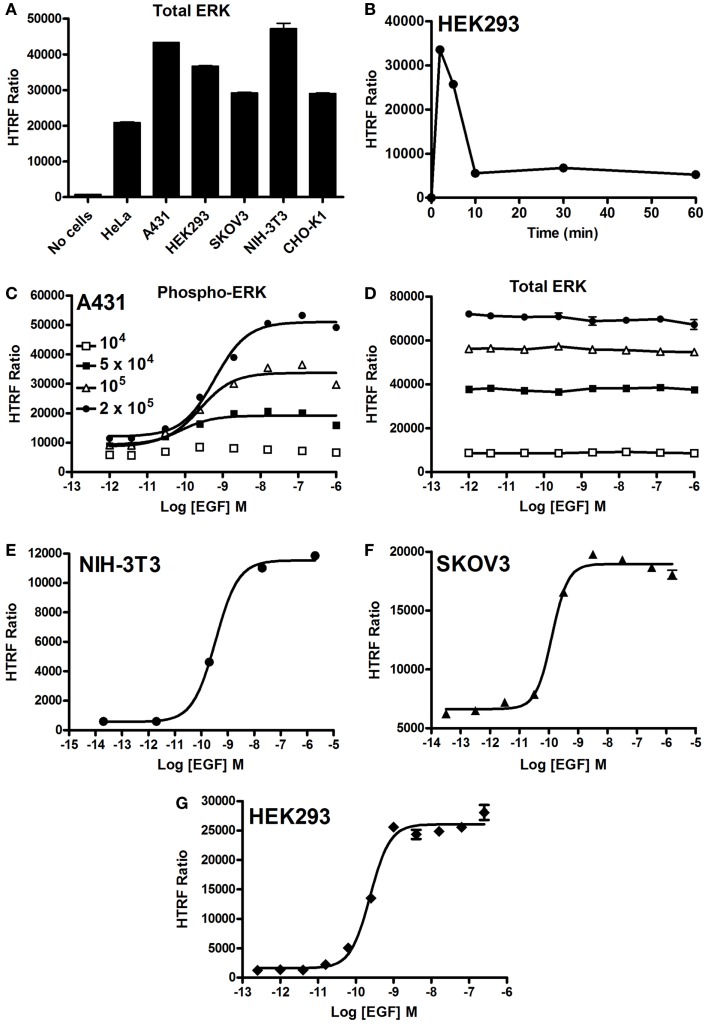
**EGF-promoted Phospho-ERK1/2 activation detected by the Phospho-ERK assay**. **(A)** Total ERK levels quantified in various cell lines. **(B)** Kinetics of EGF-induced ERK1/2 activation in HEK293 cells endogenously expressing EGFR upon their stimulation with 100 nM of EGF. **(C,D)** The effect of cell density on the phosphorylation of ERK1/2 **(C)** versus total ERK1/2 **(D)** upon stimulation with increasing concentrations of EGF as indicated. EGF-induced Phospho-ERK1/2 in various cell lines endogenously expressing EGFR: NIH-3T3 **(E)**, SKOV3 **(F)**, and HEK293 **(G)** cells (10^5^ cells/well) stimulated for 5 min with increasing concentrations of EGF before HTRF measurements were performed using the one-plate protocol. The data are mean ± SEM of three independent experiments performed in duplicate.

First, we quantified the total ERK1/2 levels in various cell lines using our HTRF-based assay and as shown in Figure 2A. Positive and specific HTRF signals reflecting the total ERK1/2 were measured and the signal varied with the cell line used. Then, we assessed the kinetics of ERK1/2 phosphorylation mediated by EGFR endogenously expressed in HEK293 cells. As shown in Figure 2B, stimulation with 100 nM EGF showed a maximal level of ERK1/2 phosphorylation at 2–5 min of stimulation, which had largely disappeared after 10 min. The transient EGF-induced ERK1/2 activation is consistent with many previous studies using different cell lines (34–36). Consequently, all the data on EGFR presented below have been generated at 5 min of stimulation with EGF. Next, we examined the effect of cell density on EGF-induced HTRF signals in the epidermal carcinoma-derived cell line A431 known as a good cell model for the study of endogenous EGFR activation and signaling. As shown in Figure 2C, the dose–response effect of EGF proportionally increased with the total number of cells per well. Such increase was consistent with the HTRF signals reflecting the total ERK1/2 expressed in the cells (Figure 2D). However, the HTRF signal resulting from total ERK1/2 was independent of EGF concentration, validating the specificity of HTRF signals as a measure of the activated ERK1/2 only (Figure 2D). Then, we examined the dose effect of EGFR-mediated ERK1/2 activation in various cell lines: NIH-3T3 mouse embryonic fibroblast (Figure 2E), SKOV3 human ovarian carcinoma (Figure 2F), and HEK293 (Figure 2G). For this, we used the one-plate protocol and cells were treated for 5 min with increasing concentrations of EGF. Such cell lines display large differences in the expression levels of EGFR per cell: 900,000 for A431 and 150,000 for SKOV3 (28), as well as 20,000 for HEK293 (data not shown). As a result, the HTRF signals reflecting ERK1/2 phosphorylation nicely increased to different extents with the increasing concentrations of EGF, and with the expected potencies (pEC_50_ values of 9.22 ± 0.11 for A431, 9.46 ± 0.06 for NIH-3T3, 9.89 ± 0.10 for SKOV3, and 9.62 ± 0.05 for HEK293) (37–39) regardless of the expression level of EGFR. Together, our data clearly demonstrate successful application of the Phospho-ERK assay to assess RTK-mediated ERK1/2 signaling in various cell models from human and mouse.

### Comparison between HTRF^®^-based and western blot-based ERK1/2 assays

**Figure 3:**
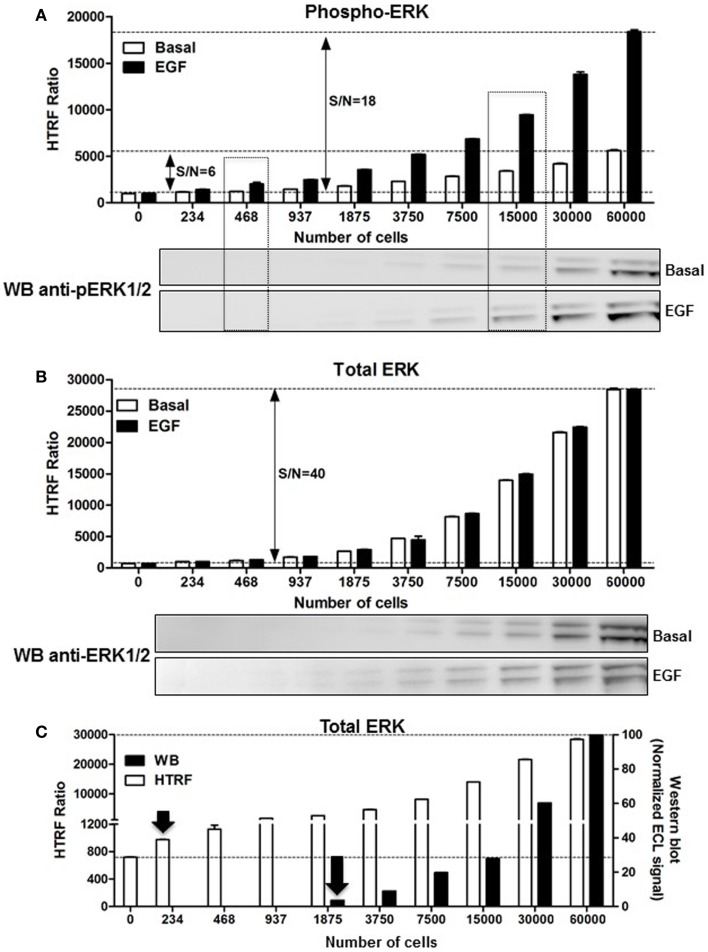
**Comparison of the Phospho-ERK and western blot assays, and effect of cell density**. A431 cells were used for the detection of the phosphorylation of ERK1/2 upon cell stimulation with 100 nM of EGF for 5 min **(A)** as well as the corresponding total ERK levels using the one-plate protocol **(B)**. S/N represents the signal-to-noise ratio through the different cell densities. For this, serial dilutions of whole cells or cell lysate were dispensed as indicated and analyzed side-by-side using the HTRF assay [top of **(A,B)**] and western blot [bottom of **(A,B)**] as described in Section “Materials and Methods.” **(C)** The detection limit of the total ERK levels using the two methods was represented by plotting the total HTRF signals (HTRF) with the enhanced chemiluminescence (ECL) signal obtained by densitometry from the western blot (WB) normalized to the signal obtained with 60,000 cells/well as 100%. The data are mean ± SEM of three independent experiments performed in duplicate.

As mentioned above, the assessment of ERK1/2 activation by western blot using an antibody recognizing the phosphorylated forms of ERK1/2 represents the widely used method. First, we performed a comparative study on A431 cells. The phosphorylation of ERK1/2 upon stimulation with 100 nM of EGF for 5 min was measured on the same number of cells comparing the western blot and HTRF^®^-based assay (Phospho-ERK assay) with the one-plate protocol. As shown in Figure 3A, the HTRF^®^-based assay used to detect the phosphorylated from of ERK1/2 presents a very good signal-to-noise ratio (S/N = 18). The Phospho-ERK assay was substantially more sensitive than the western blot assay, since ~400 cells were enough to start detecting specific EGF-induced ERK1/2 phosphorylation using the HTRF^®^-based assay while ~7500–15,000 cells were needed for the western blot method (Figure 3A). Such a significant difference in the sensitivity between the Phospho-ERK assay and western blot method was also confirmed when the total ERK1/2 levels were quantified in the different cell samples (Figure 3B). Indeed, the normalized quantification of the intensities of western blot bands of the total ERK1/2 clearly indicated that proteins were effectively detected at 1875–3750 cells while 234–468 cells per well were sufficient for the specific HTRF signal reflecting the total ERK1/2 (Figure 3C) showing a sensitivity factor of about 10-fold between the two methods.

### Use of the Phospho-ERK assay to monitor GPCR-mediated ERK1/2 activation

**Figure 4:**
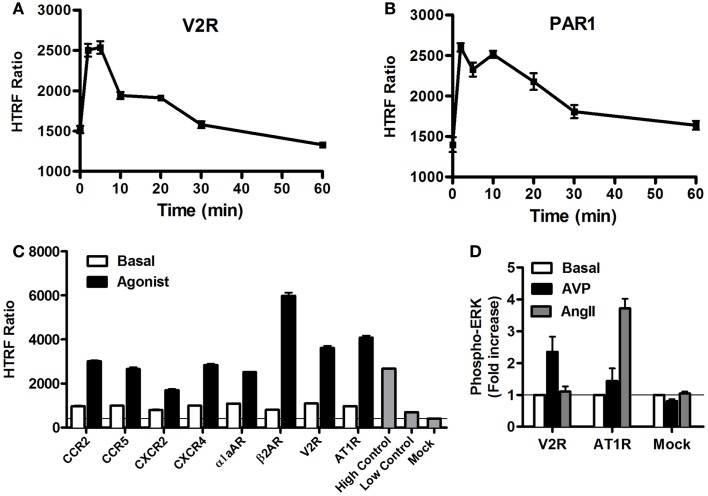
**GPCR-mediated Phospho-ERK1/2 monitored by the Phospho-ERK assay**. HEK293 cells (10^5^ cells/well) transiently expressing the different GPCRs indicated were used for the Phospho-ERK1/2 assay under basal conditions or upon cell stimulation at different time points **(A,B)** or 5 min **(C,D)** with 100 nM of the GPCR-specific agonists: AVP for V2R, thrombin (1 U/ml) for PAR1, MCP-1 for CCR2, MIP1β for CCR5, IL-8 for CXCR2, SDF1α for CXCR4, Norepinephrine for α1aAR, Isoproterenol for β2AR, and AngII for AT1R. Phospho-ERK1/2 detection was performed using the two-plate protocol, which included the additional measurement of high and low controls instead of the cell lysate. The data are mean ± SEM of three independent experiments performed in duplicate **(A–C)** or represented as fold increase in Phospho-ERK1/2 over basal and the resultant values are mean ± SEM of three independent experiments performed in triplicate **(D)**.

As stated above, it is now evident that GPCR activation can promote MAP kinase signaling, which has been shown to imply either G protein-dependent and/or G protein-independent activation pathways (4, 40, 41). Thus, we carried out a series of validation experiments with the Phospho-ERK assay on various GPCRs coupling to different heterotrimeric G proteins (Gs, Gi/o, Gq). We assessed the kinetics of ERK1/2 phosphorylation mediated by two GPCRs known to induce ERK1/2 activation, vasopressin 2 receptor (V2R) (42), and protease-activated receptor 1 (PAR1) (43, 44). The activation of V2R (Figure 4A) and PAR1 (Figure 4B), transiently expressed in HEK293 cells, with 100 nM of AVP and 1 U/ml of thrombin, respectively, induced a rapid increase in ERK1/2 phosphorylation that showed a sustained stimulation peak at 2–5 min for V2R (Figure 4A) and 2–10 min for PAR1 (Figure 4B). The activation of ERK1/2 was then followed by a slow decline to return back to the basal level after ~30 min of stimulation. The data obtained with PAR1 are in agreement with the previous observations in astrocytes using the western blot technique (44). These kinetic profiles further demonstrate GPCRs activating the mitogenic signaling pathways known to be mediated through either G protein-dependent or -independent (i.e., β-arrestin) pathways (41, 45). However, many other molecular and cellular factors have been reported to determine the duration and strength of ERK1/2 activation such as cell surface receptor density, expression of scaffolding proteins, and the balance of intracellular kinases and phosphatases (1, 7). Next, we extended our analysis to multiple other GPCRs activated for 5 min with maximal dose (100 nM) of their respective agonists using the one-plate protocol. This resulted in a substantial and specific increase in the HTRF signals compared to mock cells, with similar strength compared to the assay’s high control, thereby validating the assay (Figure 4C). To further illustrate the specificity of the GPCR agonist-induced HTRF signals, we tested the effect of cross-stimulation with 100 nM of AVP and angiotensin II (AngII) on the vasopressin V2 (V2R) and angiotensin II (AT1R) receptors transiently expressed in HEK293FT cells. As expected, AVP but not AngII specifically stimulated V2R-mediated ERK1/2 phosphorylation and *vice versa* AngII but not AVP specifically stimulated AT1R-mediated ERK1/2 phosphorylation (Figure 4D). Neither agonist promoted any ERK1/2 phosphorylation in mock-transfected cells.

### Dose–response analysis of GPCR-mediated ERK1/2 activation

**Figure 5:**
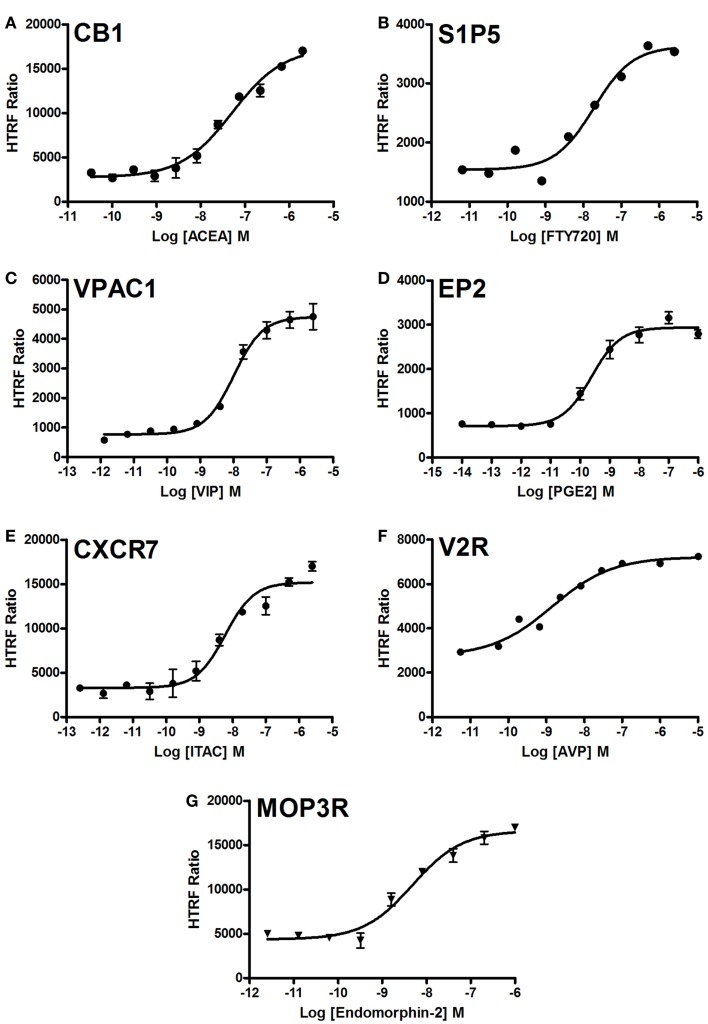
**Dose–response analysis of GPCR-mediated Phospho-ERK1/2 activation**. Agonist-induced Phospho-ERK1/2 was assessed using various GPCRs stably expressed in CHO cells: CB1 **(A)**, S1P5 **(B)**, VPAC1 **(C)**, EP2 **(D)**, CXCR7 **(E)**, V2R **(F)**, and MOP3R **(G)**. Cells were stimulated for 10 min with increasing concentrations of each GPCR-selective agonist as indicated before HTRF measurements were performed using the one-plate protocol. The figures are representative of three independent experiments performed in single points or duplicate.

We examined the dose-dependent effect of the agonists activating various GPCRs stably expressed in CHO cells and using the two-plate protocol (Figure 5). Stimulation of cells for 10 min with the GPCR-selective agonists resulted in significant dose-dependent increases in the HTRF signals to different extents. The dose–response curves exhibited the expected potencies of the agonist-receptor pairs, which are close to the nanomolar range. These data clearly demonstrate a successful application of the Phospho-ERK assay to assess the phosphorylation and activation of ERK1/2 mediated by GPCR activation regardless of the receptor’s G protein coupling profile.

### Use of the Phospho-ERK assay to monitor inhibition of receptor-mediated ERK1/2 signaling

**Figure 6:**
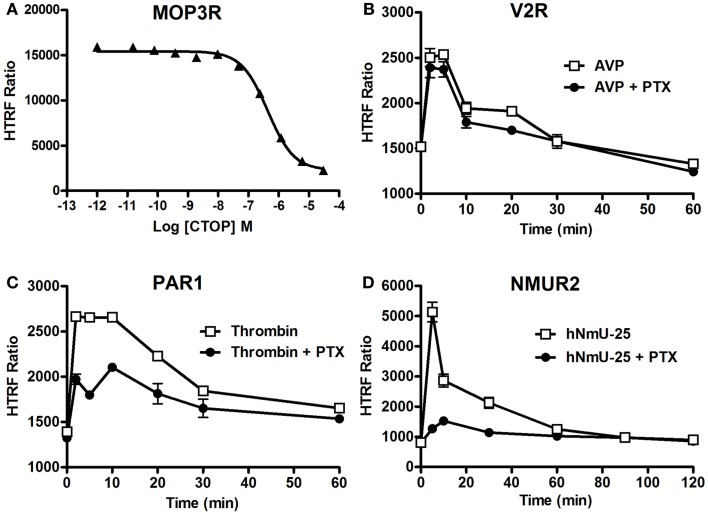
**Inhibition of ERK1/2 pathway assessed by the Phospho-ERK assay**. CHO cells stably expressing MOP3R **(A)** and HEK293 cells transiently expressing V2R **(B)**, PAR1 **(C)**, or NMUR2 **(D)** were used for the detection of ERK1/2 phosphorylation upon cell stimulation with 2 nM of DAMGO **(A)**, 100 nM of AVP **(B)**, 1 U/ml of thrombin **(C)**, or 30 nM of NmU-25 **(D)** for 5 min, using 10^5^ cells/well and the two-plate protocol. To assess the inhibition, cells were first pre-treated either with CTOP **(A)** for 15 min or overnight with 200 ng/ml of PTX **(B–D)** before agonist stimulation and HTRF measurements. The data are mean ± SEM of three independent experiments performed in duplicate.

We studied the applicability of the Phospho-ERK assay for investigating inhibitors of cell surface receptors with respect to ERK1/2. For GPCRs, we used μ-opioid receptor 3 (MOP3R) stably expressed in CHO cells as a model, as this receptor has been shown to activate ERK1/2 (46). These cells were treated with the somatostatin peptide analog (d-Phe-Cys-Tyr-d-Trp-Orn-Thr-Pen-Thr-NH2; CTOP), which has been described as a selective antagonist of μ-opioid receptors (47). As shown in Figure 6A, pre-treatment of cells for 15 min with increasing concentrations of CTOP nicely inhibited the DAMGO-induced HTRF signal, indicating the specific inhibition of ERK1/2 activation induced by MOP3R. For EGFR, both the reversible and ATP-competitive EGFR-selective inhibitor, Erlotinib (48) and the specific inhibitory anti-EGFR monoclonal antibody, Cetuximab (49), nicely inhibited the EGF-induced HTRF signal in a dose-dependent manner with the expected potencies (50, 51). These data published on the Cisbio website^3^ demonstrate the specificity of EGF-induced HTRF signals, which reflects EGFR activation promoting ERK1/2 phosphorylation.

We then examined the involvement or not of different heterotrimeric G proteins in GPCR-mediated ERK1/2 pathways since GPCRs are known to couple to different classes of heterotrimeric G proteins and to promote ERK1/2 via the activation of G protein-dependent and/or G protein-independent pathways (41, 52). Therefore, we examined the effect of pertussis toxin (PTX) blocking on GPCR-induced ERK1/2 phosphorylation using different GPCRs having different G protein coupling profiles. We tested V2R (mostly Gs) (53), PAR1 (Gi/o, Gq, and G12/13) (54), and neuromedin U receptor 2 (NMU2R; Gq and Gi/o) reported to activate ERK1/2 (55), as well as MOP3R, which is coupled to PTX-sensitive Gi/o proteins resulting in inhibition of adenylyl cyclase (56). As shown in Figure 6B, PTX did not affect the kinetics of Phospho-ERK1/2 mediated by V2R upon its stimulation with 100 nM of AVP consistent with its PTX-insensitive Gs coupling and the previous study demonstrating ERK1/2 being activated by V2R independently of heterotrimeric G protein signaling (42). In contrast, PTX treatment substantially inhibited the time-dependent ERK1/2 activation mediated by PAR1 following its stimulation with 1 U/ml of thrombin (Figure 6C). This observation is consistent with a role of PTX-sensitive G protein (i.e., Gαi/o) (54). However, the partial inhibition by PTX suggests a putative role of another PTX-insensitive G protein (i.e., Gαq) and/or G protein-independent pathway (i.e., β-arrestins) in PAR1-mediated activation of ERK1/2. For NMU2R, the ERK1/2 activation appears to be primarily mediated by a PTX-sensitive G protein since the signal peak observed at 5 min post-stimulation with 30 nM of NmU-25 was completely abolished by PTX (Figure 6D). Together, these data clearly demonstrate the successful validation of the Phospho-ERK assay to dissect the signaling pathways and the involvement of G protein-dependent and -independent signaling leading to ERK1/2 activation.

### Potential application of the Phospho-ERK assay for high-throughput screening

**Figure 7:**
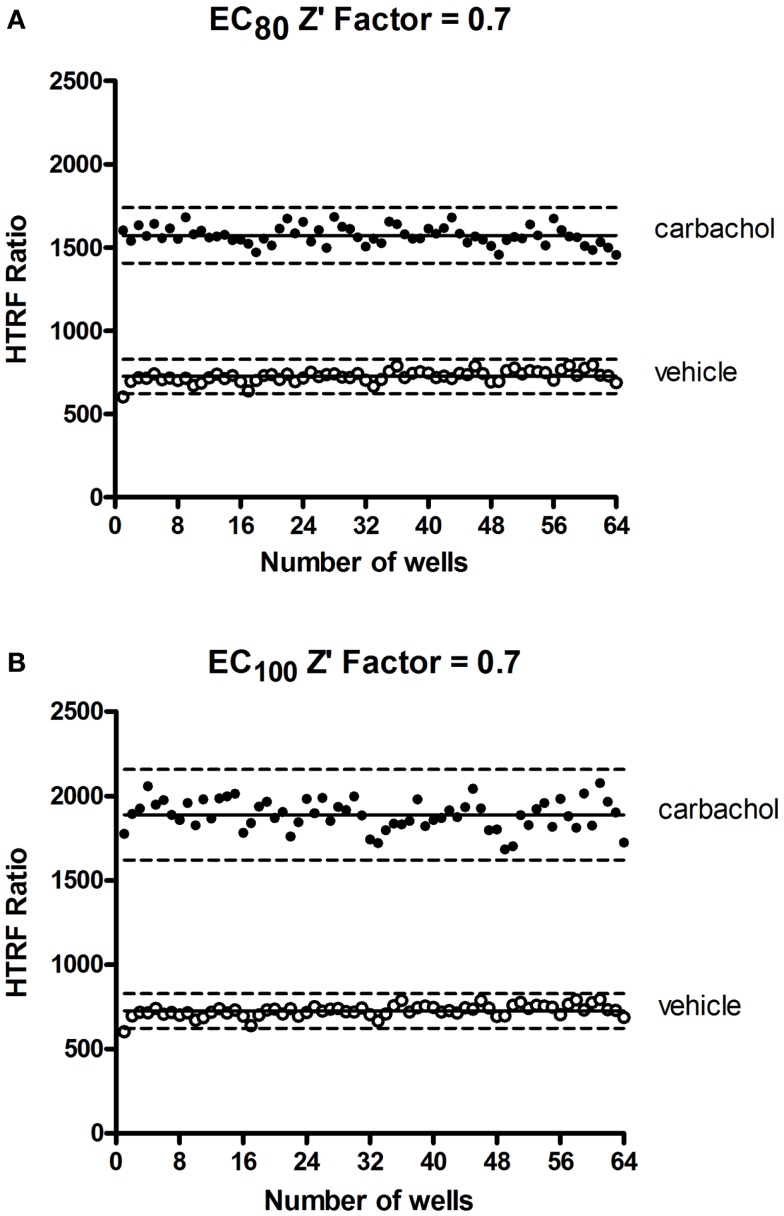
***Z*′-factor measurements for the Phospho-ERK assay**. CHO cells stably expressing the muscarinic receptor 1 (M1) were used for the determination of *Z*′-factor for the Phospho-ERK1/2 assay using the Phospho-ERK assay one-plate protocol. Cells were stimulated or not with 1.33 μM (EC_80_) **(A)** or 4 μM (EC_100_) **(B)** of carbachol for 15 min and HTRF signals were measured as described in Section “Materials and Methods.” Solid lines show the means of the positive control (carbachol) and negative control (vehicle). Broken lines display three standard deviations from the mean of each data set [Adapted from the CisBio Bioassays website (see text footnote 2) with permission].

In HTS assays, the *Z*′-factor is often used as a performance indicator for which an excess of 0.5 indicates high assay performance (57). We determined a *Z*′ value for the Phospho-ERK assay using the muscarinic receptor 1 (M1) stably expressed in CHO cells and stimulated with carbachol. The *Z*′ value of 0.7 (Figure 7) demonstrates the potential of Phospho-ERK for screening in a cell-based assay system.

In conclusion, in addition to profiling receptors and their resultant ERK1/2 signaling, the Phospho-ERK assay shows great potential for cell-based HTS. Thus, in combination with the IP-One and cAMP assays (19, 23, 24), the Phospho-ERK assay completes the innovative panel of HTRF^®^-based assays dedicated to dissecting and profiling cell surface receptor-mediated signaling (22). This is particularly powerful as it facilitates investigation of the spectrum of ligand-directed and biased signaling, with the activation of ERK1/2 constituting one of the key signaling pathways that has important physiological and pathophysiological implications.

## Author Contributions

Mohammed Akli Ayoub initiated, executed most of the experiments on GPCRs and wrote the manuscript; Julien Trebaux and Julie Vallaghe executed the experiments on EGFR; Khaled Al-Hosaini performed the experiment on NMUR2, Fabienne Charrier-Savournin, Arturo Gonzalez Moya, Jean-Philippe Pin, Kevin D. G. Pfleger, and Eric Trinquet initiated and supported the project and Kevin D. G. Pfleger also participated in the writing of the manuscript.

## Conflict of Interest Statement

Julien Trebaux, Julie Vallaghe, Fabienne Charrier-Savournin, Arturo Gonzalez Moya, and Eric Trinquet are all employees of CisBio Bioassays, which markets the HTRF Phospho-ERK kit (Catalogue number 64ERKPEG). The other co-authors report no conflicts of interest.

## Footnotes

^1^http://www.cdna.org

^2^http://www.htrf.com

^3^http://www.htrf.com/phospho-erk-thr202tyr204-cellular-assay-kit

## Abbreviations

AngII, angiotensin II; AT1R, angiotensin II type 1 receptor; AVP, arginine-vasopressin peptide; cAMP, cyclic adenosine mono-phosphate; CB1, cannabinoid receptor 1; CHO, Chinese hamster ovary; DAMGO, Tyr-Ala-Gly-*N*-methyl-Phe-Gly-ol; EGF, epidermal growth factor; EGFR, epidermal growth factor receptor; ELISA, enzyme-linked immunosorbent assay; ERK, the extracellular signal-regulated kinase; FRET, fluorescence resonance energy transfer; GPCR, G protein-coupled receptors; HEK293, human embryonic kidney 293 cells; HTRF, homogenous time-resolved fluorescence; HTS, high-throughput screening; MCP-1, monocyte chemoattractant protein 1; MIP1β, macrophage inflammatory protein 1β; MOP3R, μ-opioid receptor 3; NMU2R, neuromedin receptor 2; PAR1, protease-activated receptor 1; PGE2, prostaglandin E2; PTX, pertussis toxin; RTK, receptor tyrosine kinase; S1P5, sphingosine-1-phosphate receptor 5; SDF1α, stromal cell-derived factor 1α; V2R, vasopressin V2 receptor VIP, vasoactive intestinal peptide; VPAC1, Vasoactive intestinal polypeptide receptor 1.
